# Agreement between continuous and intermittent pulmonary artery thermodilution for cardiac output measurement in perioperative and intensive care medicine: a systematic review and meta-analysis

**DOI:** 10.1186/s13054-021-03523-7

**Published:** 2021-03-29

**Authors:** Karim Kouz, Frederic Michard, Alina Bergholz, Christina Vokuhl, Luisa Briesenick, Phillip Hoppe, Moritz Flick, Gerhard Schön, Bernd Saugel

**Affiliations:** 1 Department of Anesthesiology, Center of Anesthesiology and Intensive Care Medicine, University Medical Center Hamburg-Eppendorf, Martinistrasse 52, 20246 Hamburg, Germany; 2 MiCo, Denens, Switzerland; 3 Institute of Medical Biometry and Epidemiology, University Medical Center Hamburg-Eppendorf, Hamburg, Germany; 4 Outcomes Research Consortium, Cleveland, OH USA

**Keywords:** Cardiac index, Cardiovascular dynamics, Hemodynamic monitoring, Indicator dilution method, Pulmonary artery catheterization, Right heart catheterization, Swan-Ganz catheter

## Abstract

**Background:**

Pulmonary artery thermodilution is the clinical reference method for cardiac output monitoring. Because both continuous and intermittent pulmonary artery thermodilution are used in clinical practice it is important to know whether cardiac output measurements by the two methods are clinically interchangeable.

**Methods:**

We performed a systematic review and meta-analysis of clinical studies comparing cardiac output measurements assessed using continuous and intermittent pulmonary artery thermodilution in adult surgical and critically ill patients. 54 studies with 1522 patients were included in the analysis.

**Results:**

The heterogeneity across the studies was high. The overall random effects model-derived pooled estimate of the mean of the differences was 0.08 (95%-confidence interval 0.01 to 0.16) L/min with pooled 95%-limits of agreement of − 1.68 to 1.85 L/min and a pooled percentage error of 29.7 (95%-confidence interval 20.5 to 38.9)%.

**Conclusion:**

The heterogeneity across clinical studies comparing continuous and intermittent pulmonary artery thermodilution in adult surgical and critically ill patients is high. The overall trueness/accuracy of continuous pulmonary artery thermodilution in comparison with intermittent pulmonary artery thermodilution is good (indicated by a pooled mean of the differences < 0.1 L/min). Pooled 95%-limits of agreement of − 1.68 to 1.85 L/min and a pooled percentage error of 29.7% suggest that continuous pulmonary artery thermodilution barely passes interchangeability criteria with intermittent pulmonary artery thermodilution.

*PROSPERO registration number* CRD42020159730.

**Supplementary Information:**

The online version contains supplementary material available at 10.1186/s13054-021-03523-7.

## Background

Cardiac output (CO) monitoring is a mainstay of hemodynamic management in high-risk patients having major surgery and in critically ill patients with circulatory shock [1, 2]. Numerous technologies are available to measure or estimate CO [3–6]. Thermodilution methods allow CO calculation based on the Stewart-Hamilton principle; after injection of a known amount of indicator the change in indicator concentration downstream in the circulation is related to blood flow [7–9].

Pulmonary artery thermodilution remains the clinical reference method for CO monitoring [10]. For *intermittent* pulmonary artery thermodilution a fluid bolus with known volume and temperature is manually injected into the right atrium through the proximal port of a pulmonary artery catheter (PAC) and subsequent temperature changes over time are detected by an integrated thermistor more distal in the pulmonary artery [8]. To minimize measurement error and account for cyclic changes in CO throughout the respiratory cycle, CO is calculated based on several consecutive thermodilution CO measurements [8].

In contrast to intermittent pulmonary artery thermodilution, *continuous* pulmonary artery thermodilution enables CO to be measured automatically (i.e., without the need for manual indicator injection) [11]. PACs for continuous pulmonary artery thermodilution are equipped with a thermal filament heating up the blood in the right ventricle in a random binary sequence [11]. Changes in blood temperature are detected downstream by an integrated thermistor near the tip of the PAC. Based on the detected blood temperature changes, CO is continuously calculated using a stochastic system identification principle and an averaged CO value is provided by the monitor [11].

Because both continuous and intermittent pulmonary artery thermodilution are used in clinical practice it is important to know whether CO measurements by the two methods are clinically interchangeable. We, therefore, performed a systematic review and meta-analysis of clinical studies comparing CO measurements assessed using continuous and intermittent pulmonary artery thermodilution.

## Methods

### Study design and registration

In accordance with the Preferred Reporting Items for Systematic Reviews and Meta-Analyses (PRISMA) statement [12] we performed a systematic review and meta-analysis of clinical studies comparing continuous pulmonary artery thermodilution-derived CO measurements (CO_cont_; test method) with intermittent pulmonary artery thermodilution-derived CO measurements (CO_int_; reference method) in adult patients having surgery or critically ill patients treated in the intensive care unit. This systematic review and meta-analysis was registered in the International Prospective Register of Systematic Reviews (PROSPERO; registration number CRD42020159730).

### Eligibility criteria

For this systematic review and meta-analysis, we considered studies published in English between January 1st, 1975 and December 31st, 2019 comparing CO_cont_ and CO_int_ in adult (age ≥ 18 years) surgical or critically ill patients that report extractable or calculable mean of the differences between CO_cont_ and CO_int_ with corresponding standard deviation (SD) and/or 95%-limits of agreement (95% LOA). We did not consider correspondences or case reports.

### Information sources and search strategy

The electronic databases PubMed, Web of Science, and the Cochrane Library were systematically searched using a priori defined search strategies. As an example, the full electronic search strategy for PubMed is provided in Additional file 1. Further, the reference lists of the identified studies and the reference lists of previous reviews were searched to find additional eligible studies that had not been identified during the initial systematic database search.

### Study selection

Titles and abstracts of all identified studies were screened by three investigators (PH, MF, BS). The full-text of potentially eligible studies was used to assess study eligibility based on the above-mentioned predefined eligibility criteria. Discrepancies were resolved by discussion among the three investigators.

### Data collection process and data items

Four different investigators (KK, AB, CV, LB) independently extracted the data from the included studies and data were checked for consistency. Discrepancies were discussed and resolved based on the original data. We extracted data on the results of comparative statistics, i.e., the mean of the differences between CO_cont_ and CO_int_ with SD, 95% LOA, and the percentage error (PE) [13]. We report the mean of the differences between CO_cont_ and CO_int_ as CO_cont_ − CO_int_. We re-calculated the mean of the differences for studies reporting the mean of the differences as CO_int_ − CO_cont_ accordingly. If not provided in the studies, the SD of the mean of the differences was re-calculated as (upper 95% LOA − mean of the differences)/1.96. For studies not providing the PE but reporting mean CO_cont_ and mean CO_int_, the PE was calculated as (1.96 **⋅** SD of the mean of the differences)/(mean of CO_cont_ and CO_int_).

In addition to the results of comparative statistics, we extracted data regarding the study setting (operating room or intensive care unit), the patient population, the number of patients, the total number of measurement pairs, and the year of publication.

### Risk of bias in individual studies

Based on the Quality Assessment of Diagnostic Accuracy Studies guidelines (QUADAS-2) [14] we used an adapted questionnaire (Additional file 2) to assess study quality by objectively performing judgments on bias and applicability of the included studies [14–16]. Risk of bias classification is based on different signaling questions of different domains that were marked with “yes”, “no” or “unclear” which finally results in classifying these domains as “low”, “high” or “unclear” risk of bias. Concerns about applicability of the included studies were rated as “low”, “high” or “unclear”. An independent quality assessment of each included study was performed by three investigators (KK, AB, LB) and discrepancies were resolved by discussion among the three investigators.

### Principle summary measures

The mean of the differences between CO_cont_ and CO_int_ of the individual studies is the principal summary measure of the current meta-analysis. We used a random effects model for means as outcomes with restricted maximum likelihood as the estimator to summarize the mean of the differences, the SD of the mean of the differences, and the sample size. This random effects model derives a pooled estimate of the mean of the differences that represents the trueness/accuracy of CO_cont_ compared to CO_int_.

For each study, we calculated the 95%-confidence interval (95% CI) for the reported/calculated mean of the differences between CO_cont_ and CO_int_ as 1.96 **⋅** standard error of the mean (SD/√sample size) to account for study sample size. We summarized these 95% CIs with the random effects model and report the resulting overall random effects model-derived pooled estimate of the 95% CI.

Further, we report overall random effects model-derived pooled estimates of 95% LOA.

We summarized the PE using a random effects model for proportions with DerSimonian-Laird as the estimator [17] and report the overall random effects model-derived pooled estimate of the PE with 95% CI. We defined clinical interchangeability between CO_cont_ and CO_int_ based on the established 30% PE threshold [13]. Heterogeneity and inconsistency were assessed by means of Cochran’s Q and I^2^.

### Synthesis of results

The database includes all relevant data to perform the meta-analysis. To obtain overall random effects model-derived pooled estimates, a random effects model was computed for each outcome. We reported Cochran’s Q as a measure of heterogeneity and I^2^ as a measure of consistency.

### Risk of publication bias across studies

We calculated funnel plots with corresponding Eggers regression tests for asymmetry to address the potential problem of selective reporting [18].

### Subgroup analyses, additional analyses

We performed subgroup analyses considering the factors "setting" (operating room and intensive care unit) and “patient population” (liver transplantation and cardiac surgery).

Additionally, we investigated the relation between the mean of the differences between CO_cont_ and CO_int_ from individual studies and a) the reported mean CO_int_ and b) the year of publication.

### Statistical software

We used the software R version 4.0.2 (R Foundation for Statistical Computing. Vienna, Austria) with the R-package metafor version 2.4–0 for statistical analyses [19].

## Results

### Study selection

**Fig. 1:**
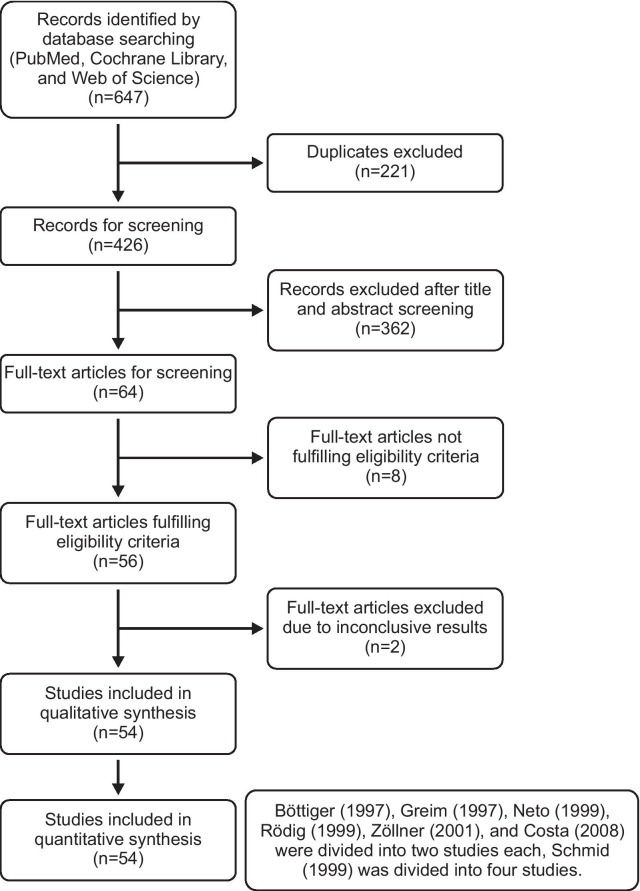
Flowchart of the literature search based on the PRISMA statement

After removal of duplicates, we identified 426 different records based on the initial electronic database search (Fig. 1). We excluded 362 records after title and abstract screening. Full-text screening of the remaining 64 articles identified 54 studies fulfilling our predefined inclusion criteria [20–73]. Six studies were divided into two studies each for the following reasons: measurements before and after caval clamping/graft perfusion during liver transplantation [26], measurements reported separately for infusion rates > 1000 mL/h and $\le$ 1000 mL/h [41], measurements with different PAC devices [60, 72], measurements reported separately for patients with an ejection fraction higher or lower than 45% [65], and measurements reported separately for patients with a CO higher or lower than 8 L/min [36]. One study was divided into four studies because different software versions and different fluid bolus temperatures were used [67].

### Study characteristics

We included a total number of 1,522 individual patients in the final analysis with a median of 21 patients included per study (minimum: 7 patients, maximum: 84 patients). All studies reported the number of measurement pairs except for one study. The total number of reported measurement pairs was 17,920 with a median of 168 (interquartile range 108 and 238) measurement pairs per study. In 51 of the 54 studies, the mean of the differences was reported; for the remaining three studies the mean of the differences was calculated. In 24 of the 54 studies, 95% LOA were reported; for 30 studies 95% LOA were calculated. In 11 of the 54 studies, the PE was reported; for 16 studies the PE was calculated. In 23 of the 54 studies, the mean values of CO_cont_ and CO_int_ were reported or calculated. A summary of the included studies and CO measurement data is provided in Additional file 3.

### Risk of bias in individual studies

The adapted QUADAS-2 questionnaire was used to assess the risk of bias in the included studies (Additional file 4). In 19 studies, the risk of bias was identified to be “unclear” or “high” at least for one domain, in six studies, the risk of bias was identified to be “high” at least for one domain.

### Overall meta-analysis

**Fig. 2:**
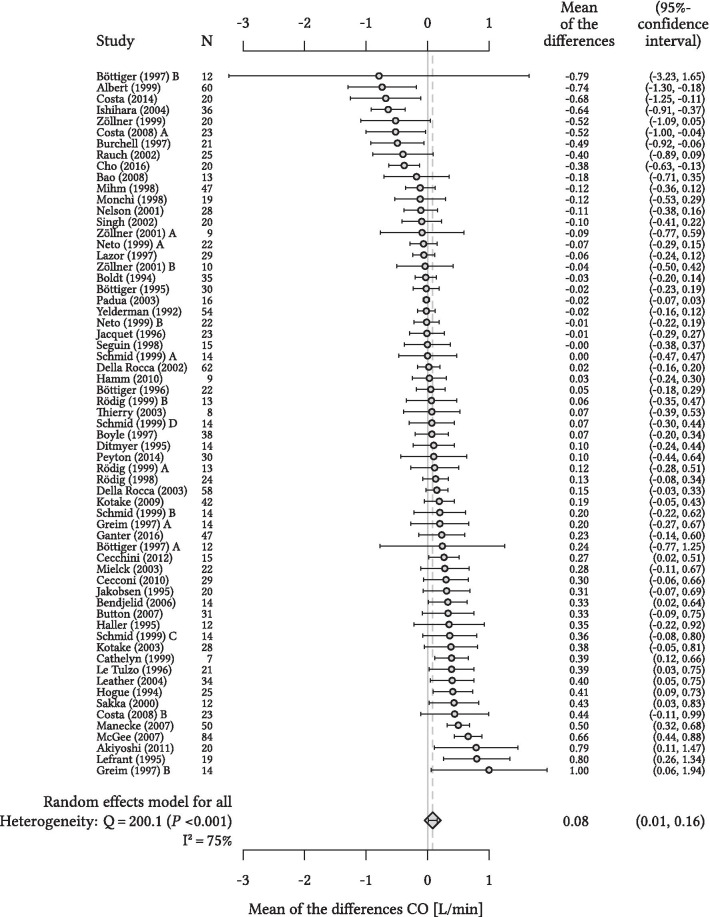
Forest plot for cardiac output. Forest plot showing the results of the meta-analysis for cardiac output (CO) with mean of the differences (dots) calculated as the mean of continuous pulmonary artery thermodilution-derived CO measurements minus intermittent pulmonary artery thermodilution-derived CO measurements and corresponding 95%-confidence interval (bars) per individual study in relation to the overall random effects model-derived pooled estimate (vertical dashed line). Heterogeneity is presented with Cochran’s Q and I^2^. N, number of patients per study. Böttiger and colleagues [26], Costa and colleagues [36], Greim and colleagues [41], Neto and colleagues [60], Rödig and colleagues [65], and Zöllner and colleagues [72] are treated as two studies in the analysis (A and B). Schmid and colleagues [67] is treated as four studies in the analysis (A, B, C, and D)

Individual means of the differences between CO_cont_ and CO_int_ with SD and 95% LOA for each study are shown in Additional file 3. The overall random effects model-derived pooled estimate of the mean of the differences between CO_cont_ and CO_int_ was 0.08 (95% CI 0.01 to 0.16) L/min with pooled 95% LOA of − 1.68 to 1.85 L/min (heterogeneity: Q = 200.1 (*P* < 0.001), I^2^ = 75%) (Fig. 2).

**Fig. 3:**
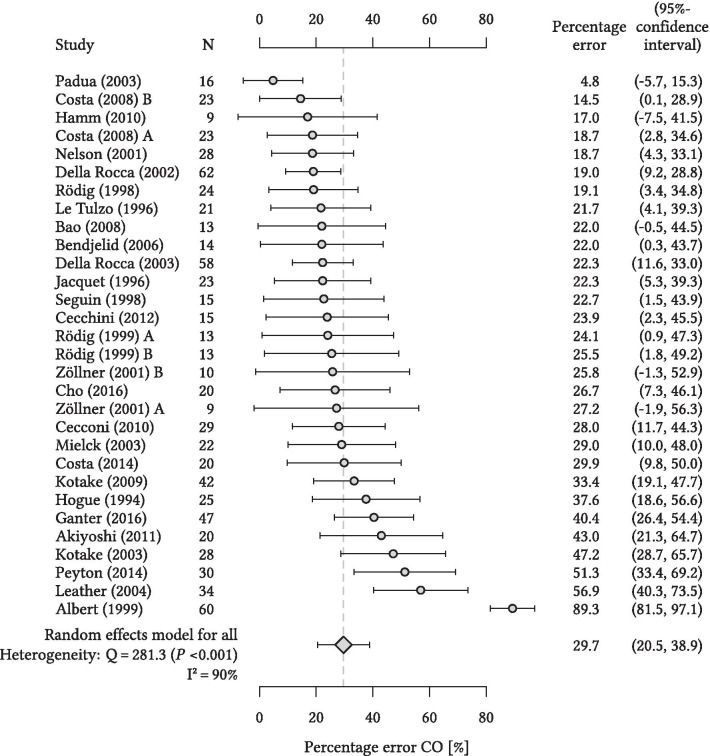
Forest plot for percentage error. Forest plot showing the results of the meta-analysis for the percentage error (dots) with 95%-confidence interval (bars) per individual study in relation to the overall random effects model-derived pooled estimate (vertical dashed line). Heterogeneity is presented with Cochran’s Q and I^2^. CO, cardiac output; N, number of patients per study. Costa and colleagues [36], Rödig and colleagues [65], and Zöllner and colleagues [72] are treated as two studies in the analysis (A and B)

The overall random effects model-derived pooled estimate of the PE was 29.7% with 95% CI of 20.5 to 38.9% (heterogeneity: Q = 281.3 (*P* < 0.001), I^2^ = 90%) (Fig. 3). The PE was ≤ 30% in 19 out of 27 studies (70%).

### Risk of publication bias across studies

Funnel plots indicating the risk of publication bias across studies including Eggers regression tests are shown in Additional file 5 for CO (*P* = 0.843), and Additional file 6 for PE (*P* = 0.474).

### Subgroup analyses, additional analyses

We performed subgroup analyses considering the factors "setting" (operating room and intensive care unit), “patient population” (liver transplantation and cardiac surgery), and “availability of the PE” (studies where the PE was reported or calculable and studies where the PE was not reported or calculable).

For patients studied in the operating room [20, 22, 26, 34, 37, 38, 41, 44, 48, 49, 52, 62, 64, 69], the overall random effects model-derived estimate of the mean of the differences was 0.14 (95% CI 0.00 to 0.28) L/min with pooled 95% LOA of − 2.03 to 2.44 L/min (Additional file 7). For patients studied in the intensive care unit [21, 23, 24, 27–29, 31–33, 35, 36, 39, 40, 42, 45, 46, 51, 53–60, 66–68, 70–73], the overall random effects model-derived estimate of the mean of the differences was 0.07 (95% CI − 0.04 to 0.17) L/min with pooled 95% LOA of − 1.66 to 1.76 L/min (Additional file 8).

For patients having liver transplantation [20, 22, 26, 35, 36, 38, 41], the overall random effects model-derived estimate of the mean of the differences was 0.07 (95% CI − 0.26 to 0.40) L/min with pooled 95% LOA of − 2.89 to 3.01 L/min (Additional file 9). For patients having cardiac surgery [23, 25, 27, 30–32, 34, 39, 43–45, 47–49, 52, 54, 56, 60, 61, 63–65, 67, 69, 70, 72, 73], the overall random effects model-derived estimate of the mean of the differences was 0.09 (95% CI − 0.01 to 0.18) L/min with pooled 95% LOA of − 1.38 to 1.54 L/min (Additional file 10).

There were no clinically meaningful differences in the mean of the differences and the 95% LOA between studies with reported/calculable PE and studies without reported/calculable PE (Additional files 11 and 12).

The mean of the differences between CO_cont_ and CO_int_ from individual studies was not influenced by the reported mean CO_int_ (Additional file 13) or the year of publication (Additional file 14).

## Discussion

In this meta-analysis of clinical studies comparing CO_cont_ and CO_int_ in adult surgical and critically ill patients, the heterogeneity across studies was high. The overall random effects model-derived pooled estimate of the mean of the differences between CO_cont_ and CO_int_ was 0.08 L/min with pooled 95% LOA of − 1.68 to 1.85 L/min and a pooled PE of 29.7 (95% CI 20.5 to 38.9)%.

In CO method comparison studies, the agreement between a test and a reference method is described by the trueness (often called “accuracy”) and precision of agreement [74–76] based on Bland–Altman analysis [77–79]. In Bland–Altman plots, the difference between measurements with a test and a reference method is plotted against the mean of the two measurements [77–79]. The mean of the differences (often called “bias”) reflects the trueness of test method measurements, the SD and 95% LOA of the mean of the differences reflect the precision of agreement [74–76]. The PE is used frequently in CO method comparison studies to characterize the precision of agreement; the PE is 1.96 SD of the mean of the differences between measurements divided by the mean value of all measurements [13]. In their landmark study, Critchley et al. proposed 28.3%, rounded up to 30%, as the PE threshold defining interchangeability [13]. Nevertheless, one should keep in mind that the PE threshold of 28.3% is based on the assumption that the precision of method of both the test method and the reference method are 20%. Because the precision of method is not exactly known, using a 30% PE threshold may lead to misinterpretations concerning the clinical interchangeability of CO_cont_ and CO_int_.

In this meta-analysis, the overall random effects model-derived pooled estimate of the mean of the differences between CO_cont_ and CO_int_ was < 0.1 L/min—which is less than a 2% difference for an average adult CO of 5 to 6 L/min. This meta-analysis thus suggests a good trueness/accuracy of CO_cont_ compared with CO_int_ when looking at the overall pooled mean of the differences. However, a low pooled mean of the differences in meta-analyses can be misleading because averaging study results with negative and positive means of the differences of similar absolute amount can result in a very low pooled mean of the differences despite marked measurement differences in single studies. In this meta-analysis, studies reporting an overestimation and those reporting an underestimation of CO_cont_ compared to CO_int_ neutralized each other, as illustrated in Fig. 2.

Regarding the precision of agreement between CO_cont_ and CO_int_ this meta-analysis revealed that the pooled 95% LOA of the mean of the differences between CO_cont_ and CO_int_ were − 1.68 to 1.85 L/min. The overall random effects model-derived pooled estimate of the PE was 29.7 (95% CI 20.5 to 38.9)%—thus suggesting that CO_cont_ barely passes interchangeability criteria with CO_int_ [13]. However, the PE was only available for half of all studies because the PE per se or mean CO values necessary for post-hoc PE calculation were not always reported. Nevertheless, 95% CIs were similar in studies with reported or calculable PE and studies where the PE was not reported or calculable suggesting that the PE for all studies would probably also be close to 30%.

This meta-analysis showed a large variability in results between studies, with means of the differences reported in single studies ranging from − 0.79 to 1.00 L/min and PEs ranging from 4.8 to 89.3%. This variability strongly suggests that the measurement performance of CO_cont_ is influenced by various factors, that may include patient characteristics, the clinical setting, and cardiovascular dynamics. Even subgroups of studies were heterogeneous. For example, the “operating room” subgroup included patients having different types of surgery, the “intensive care unit” subgroup included patients with and without circulatory shock requiring different vasopressor and inotropic support, and the “cardiac surgery” subgroup included patients studied either during or after surgery. It is important to bear in mind that the measurement performance is context-sensitive when interpreting validation studies of any CO monitoring system [80].

Intermittent pulmonary artery thermodilution remains the clinical reference method for CO monitoring and therefore is frequently used as the “gold standard” in method comparison studies [10]. Continuous pulmonary artery thermodilution offers the opportunity to measure CO automatically without the need for manual indicator injection, thus reducing contamination risk and saving time [81]. Although “continuous” suggests that this PAC technology provides real-time CO measurements, it actually provides “semi-continuous”, averaged CO values [11, 81]. The averaging procedure improves the signal-to-noise ratio but may cause a time delay of up to several minutes. This time delay may become relevant when hemodynamics change rapidly, e.g., during dynamic tests such as passive leg raising and during therapeutic interventions such as fluid or vasopressor administration [8, 82, 83].

In today’s clinical practice, PACs are mainly used in patients having cardiac surgery, liver transplantation, and in critically ill patients with circulatory shock, especially with right ventricular dysfunction [10, 84]. Using a PAC allows monitoring of CO, mixed venous oxygen saturation, and intravascular pressure and thus provides important information on cardiovascular dynamics [85]. There is nonetheless an ongoing debate on whether or not PACs still have a place in daily clinical practice [86–88]. Some trials showed no clinical benefit of using the PAC without treatment protocols in critically ill patients [89, 90] or cardiac surgery patients [91]. Additionally, there are now various methods to measure or estimate CO less invasively or even non-invasively [3, 6]. The clinical use of the PAC thus decreased over the last years in critically ill patients and in surgical patients [92, 93].

Although intermittent and continuous pulmonary thermodilution methods are widely used, we are not aware of any meta-analysis investigating the overall agreement between the two methods. In contrast, several meta-analyses have already been published for Doppler [94, 95], bioimpedance [15, 94], as well as invasive and non-invasive pulse contour methods [15, 16, 94]. They all reported pooled PE values ranging between 40 and 50%.

We only investigated the absolute agreement between CO_cont_ and CO_int_ and did not analyze the trending ability of CO_cont_. The ability to track changes in CO is actually the main expectation clinicians may have from a continuous monitoring system over an intermittent technique. Unfortunately, most studies of this meta-analysis did not report concordance rates or polar plots, so that we were unable to assess the ability of continuous pulmonary thermodilution to track changes in CO. Furthermore, several studies [19 of 54 (35%)] had a risk of bias classification of “unclear” or “high” that may further influence the final results of this meta-analysis. About half of the included studies [26 of 54 (48%)] were performed before the year 2000, and only 6 (11%) studies after 2010.

## Conclusion

The heterogeneity across clinical studies comparing CO_cont_ and CO_int_ in adult surgical and critically ill patients is high. The overall trueness/accuracy of CO_cont_ in comparison with CO_int_ is good (indicated by a pooled mean of the differences < 0.1 L/min). Pooled 95% LOA of − 1.68 to 1.85 L/min and a pooled PE of 29.7 (95% CI 20.5 to 38.9)% suggest that CO_cont_ barely passes interchangeability criteria with CO_int_. The PE was ≤ 30% in two-thirds of studies with available PE.

## Supplementary Information

**Additional file 1**. Electronic search strategy for PubMed. This file contains the full electronic search strategy for PubMed.

**Additional file 2**. Adapted QUADAS-2 questionnaire. This file contains the adapted QUADAS-2 questionnaire that was used to assess study quality by objectively performing judgments on bias and applicability of the included studies.

**Additional file 3**. Summary of the included studies and cardiac output measurement data. This file contains a table summarizing the included studies and extracted cardiac output measurement data.

**Additional file 4**. Risk of bias assessment. This file contains the results of the risk of bias assessment of all included studies.

**Additional file 5**. Funnel plot for cardiac output with Eggers regression test. Funnel plot indicating the risk of publication bias across studies including Eggers regression for cardiac output (CO).

**Additional file 6**. Funnel plot for the percentage error with Eggers regression test. Funnel plot indicating the risk of publication bias across studies including Eggers regression for the percentage error. CO, cardiac output.

**Additional file 7**. Forest plot showing subgroup analysis for the setting “operating room”. Forest plot showing the results of the subgroup analysis for the setting “operating room” for cardiac output (CO) with mean of the differences (dots) and corresponding 95%-confidence interval (bars) per individual study in relation to the overall random effects model-derived pooled estimate (vertical dashed line). Heterogeneity is presented with Cochran’s Q and I^2^. N, number of patients per study. Böttiger and colleagues [26], and Greim and colleagues [41] are treated as two studies in the analysis (A and B).

**Additional file 8**. Forest plot showing subgroup analysis for the setting “intensive care unit”. Forest plot showing the results of the subgroup analysis for the setting “intensive care unit” for cardiac output (CO) with mean of the differences (dots) and corresponding 95%-confidence interval (bars) per individual study in relation to the overall random effects model-derived pooled estimate (vertical dashed line). Heterogeneity is presented with Cochran’s Q and I^2^. N, number of patients per study. Costa and colleagues [36], Neto and colleagues [60], and Zöllner and colleagues [72] are treated as two studies in the analysis (A and B). Schmid and colleagues [67] is treated as four studies in the analysis (A, B, C, and D).

**Additional file 9**. Forest plot showing subgroup analysis for patients having liver transplantation. Forest plot showing the results of the subgroup analysis for patients having liver transplantation for cardiac output (CO) with mean of the differences (dots) and corresponding 95%-confidence interval (bars) per individual study in relation to the overall random effects model-derived pooled estimate (vertical dashed line). Heterogeneity is presented with Cochran’s Q and I^2^. N, number of patients per study. Böttiger and colleagues [26], Costa and colleagues [36], and Greim and colleagues [41] are treated as two studies in the analysis (A and B).

**Additional file 10**. Forest plot showing subgroup analysis for patients having cardiac surgery. Forest plot showing the results of the subgroup analysis for patients having cardiac surgery for cardiac output (CO) with mean of the differences (dots) and corresponding 95%-confidence interval (bars) per individual study in relation to the overall random effects model-derived pooled estimate (vertical dashed line). Heterogeneity is presented with Cochran’s Q and I^2^. N, number of patients per study. Neto and colleagues [60], Rödig and colleagues [65], and Zöllner and colleagues [72] are treated as two studies in the analysis (A and B). Schmid and colleagues [67] is treated as four studies in the analysis (A, B, C, and D).

**Additional file 11**. Forest plot showing subgroup analysis for studies with reported or calculable percentage error. Forest plot showing the results of the subgroup analysis for studies with reported or calculable percentage error for cardiac output (CO) with mean of the differences (dots) and corresponding 95%-confidence interval (bars) per individual study in relation to the overall random effects model-derived pooled estimate (vertical dashed line). Heterogeneity is presented with Cochran’s Q and I^2^. N, number of patients per study. Costa and colleagues [36], Rödig and colleagues [65], and Zöllner and colleagues [72] are treated as two studies in the analysis (A and B).

**Additional file 12**. Forest plot showing subgroup analysis for studies without reported or calculable percentage error. Forest plot showing the results of the subgroup analysis for studies without reported or calculable percentage error for cardiac output (CO) with mean of the differences (dots) and corresponding 95%-confidence interval (bars) per individual study in relation to the overall random effects model-derived pooled estimate (vertical dashed line). Heterogeneity is presented with Cochran’s Q and I^2^. N, number of patients per study. Böttiger and colleagues [26], Greim and colleagues [41], and Neto and colleagues [60], are treated as two studies in the analysis (A and B). Schmid and colleagues [67] is treated as four studies in the analysis (A, B, C, and D).

**Additional file 13**. Influence of the level of cardiac output. Plot showing the relation between the mean of the differences (dots) with corresponding 95%-confidence interval (bars) per individual study and mean intermittent pulmonary artery catheter-derived cardiac output (CO) measurement (CO_int_).

**Additional file 14**. Influence of the year of publication. Plot showing the relation between the mean of the differences (dots) with corresponding 95%-confidence interval (bars) per individual study and the year of publication. CO, cardiac output.

## Data Availability

The datasets used and/or analyzed during the current study are available from the corresponding author on reasonable request.

## Abbreviations

95% CI: 95%-confidence interval
95% LOA: 95%-limits of agreement
CO: Cardiac output
CO_cont_: Continuous pulmonary artery thermodilution-derived CO measurements
CO_int_: Intermittent pulmonary artery thermodilution-derived CO measurements
PAC: Pulmonary artery catheter
PE: Percentage error
PRISMA: Preferred Reporting Items for Systematic Reviews and Meta-Analyses
PROSPERO: International Prospective Register of Systematic Reviews
QUADAS: Quality Assessment of Diagnostic Accuracy Studies guidelines
SD: Standard deviation
