# Supplementary material for: Agreement between continuous and intermittent pulmonary artery thermodilution for cardiac output measurement in perioperative and intensive care medicine: a systematic review and meta-analysis
Source: Crit Care. 2021 Mar 29;25:125. doi: 10.1186/s13054-021-03523-7 (PMC8006374; doi:10.1186/s13054-021-03523-7)
Supplement: Supplementary file 1 — Additional file 1. Electronic search strategy for PubMed. This file contains the full electronic search strategy for PubMed. [file 13054_2021_3523_MOESM1_ESM.pdf]

Additional file 1: Electronic search strategy for PubMed

1. cardiac output OR cardiac index
2. NOT neonates OR neonate OR neonatal OR newborn OR newborns OR infants OR infant OR child OR children OR pediatric OR pediatrics OR paediatric OR paediatrics OR animals OR animal
3. AND continuous OR semi-continuous OR semicontinuous
4. AND thermodilution OR bolus
5. AND bias OR mean difference OR limit of agreement OR limits of agreement OR LOA OR Bland Altman OR Bland-Altmann OR standard deviation OR precision OR accuracy OR percentage error
6. AND critical care OR critically ill OR intensive care unit OR “intensive care” OR ICU OR surgery OR surgical OR operation OR operative OR procedure OR operating OR preoperative OR pre-operative OR peri-operative OR perioperative OR intra-operative OR intraoperative OR post-operative OR postoperative OR anesthesia OR anaesthesia OR anesthesiology OR anaesthesiology

((((((cardiac output OR cardiac index)) NOT (neonates OR neonate OR neonatal OR newborn OR newborns OR infants OR infant OR child OR children OR pediatric OR pediatrics OR paediatric OR paediatrics OR animals OR animal)) AND (continuous OR semi-continuous OR semicontinuous)) AND (thermodilution OR bolus)) AND (bias OR mean difference OR limit of agreement OR limits of agreement OR LOA OR Bland Altman OR Bland-Altmann OR standard deviation OR precision OR accuracy OR percentage error)) AND (critical care OR critically ill OR intensive care unit OR “intensive care” OR ICU OR surgery OR surgical OR operation OR operative OR procedure OR operating OR preoperative OR pre-operative OR peri-operative OR perioperative OR intra-operative OR intraoperative OR post-operative OR postoperative OR anesthesia OR anaesthesia OR anesthesiology OR anaesthesiology)

Filters: English language, Humans
