# Supplementary material for: Agreement between continuous and intermittent pulmonary artery thermodilution for cardiac output measurement in perioperative and intensive care medicine: a systematic review and meta-analysis
Source: Crit Care. 2021 Mar 29;25:125. doi: 10.1186/s13054-021-03523-7 (PMC8006374; doi:10.1186/s13054-021-03523-7)
Supplement: Supplementary file 2 — Additional file 2. Adapted QUADAS-2 questionnaire. This file contains the adapted QUADAS-2 questionnaire that was used to assess study quality by objectively performing judgments on bias and applicability of the included studies. [file 13054_2021_3523_MOESM2_ESM.pdf]

Additional file 2: Adapted QUADAS-2 questionnaire

Article Number	Author	Date	Reviewer

Risk of bias is judged as “low,” “high,” or “unclear.” If the answers to all signaling questions for a domain are “yes,” then risk of bias can be judged low. If any signaling question is answered “no,” potential for bias exists. Review authors must then use the guidelines developed in phase 2 to judge risk of bias. The “unclear” category should be used only when insufficient data are reported to permit a judgment.			
**Domain 1: Patient selection**			
Risk of Bias: Could the selection of patients have introduced bias?<br>(Two No = High)	Low	High	Unclear
Signaling question 1: Did the study describe population of interest and demographic data (e.g., sex, age, underlying disease(s))?	Yes	No	Unclear
Signaling question 2: Were the inclusion and exclusion criteria clearly described?	Yes	No	Unclear
Applicability: Are there concerns that the included patients and setting do not match the review question?	Low	High	Unclear
**Domain 2: Index test (continuous cardiac output monitoring)**			
Risk of bias: Could the conduct or interpretation of continuous cardiac output monitoring have introduced bias? (No = High)	Low	High	Unclear
Signaling question: Was the setting of continuous cardiac output monitoring described clearly? (e.g., type of pulmonary artery catheter (PAC), type of monitor, correct PAC placement, etc.)	Yes	No	Unclear
Applicability: Are there concerns that the index test, its conduct, or its interpretation differ from the review question?	Low	High	Unclear
**Domain 3: Reference standard (intermittent cardiac output monitoring)**			
Risk of bias: Could the reference method, its conduct, or its interpretation have introduced bias? (No = High)	Low	High	Unclear
Signaling question: Is the reference cardiac output monitoring likely to correctly measure cardiac output (e.g., type of PAC, type of monitor, correct PAC placement, description of bolus application)?	Yes	No	Unclear
Applicability: Is there concern that the target condition as defined by the reference standard does not match the review question?	Low	High	Unclear
**Domain 4: Flow and timing**			
Could the patient flow have introduced bias? Could the analysis of flow and timing have introduced bias? (Unclear: ≥2 unclear, 1 No + 1 unclear) (High: ≥2 No)	Low	High	Unclear
Signaling question 1: Were the number of patients enrolled and who dropped out clearly described in the result (e.g., number of enrolled patients = reported number of patients in results section OR drop out clearly described)?	Yes	No	Unclear
Signaling question 2: Were the reference and index test measured “simultaneously” (directly before/after)?	Yes	No	Unclear
Signaling question 3: Was the method of acquiring paired measurements well described?	Yes	No	Unclear
Signaling question 4: In case of repeated measurements of cardiac output in patients, did they use statistical analysis for agreement between methods of measurement with multiple observations per individual?	Yes	No	Unclear
Signaling question 5: In case of the mean of the differences being described in both the article and the figure(s), do they match?	Yes	No	Unclear
