# Supplementary material for: Agreement between continuous and intermittent pulmonary artery thermodilution for cardiac output measurement in perioperative and intensive care medicine: a systematic review and meta-analysis
Source: Crit Care. 2021 Mar 29;25:125. doi: 10.1186/s13054-021-03523-7 (PMC8006374; doi:10.1186/s13054-021-03523-7)
Supplement: Supplementary file 3 — Additional file 3. Summary of the included studies and cardiac output measurement data. This file contains a table summarizing the included studies and extracted cardiac output measurement data. [file 13054_2021_3523_MOESM3_ESM.pdf]

Additional file 3: Summary of the included studies and cardiac output measurement data

First author	Year	Journal	Setting	Patient population	Patients (*n*)	MD (SD) (L/min)	Lower 95%-LOA (L/min)	Upper 95%-LOA (L/min)	PE (%)
Yelderman	1992	*J Cardiothorac Vasc Anesth*	ICU	critically ill	54	-0.02 (0.54)	-1.07	1.03	na
Boldt	1994	*Crit Care Med*	ICU	critically ill	35	-0.03 (0.52)	-1.07	1.01	na
Hogue	1994	*J Cardiothorac Vasc Anesth*	OR	cardiac surgery	25	0.41 (0.82)	-1.23	2.05	37.6
Böttiger	1995	*J Cardiothorac Vasc Anesth*	OR/ICU	cardiac surgery	30	-0.02 (0.59)	-1.18	1.14	na
Ditmyer	1995	*Am J Crit Care*	ICU	cardiac surgery	14	0.10 (0.64)	-1.18	1.34	na
Haller	1995	*Crit Care Med*	ICU	critically ill	12	0.35 (1.01)	-1.67	2.37	na
Jakobsen	1995	*Acta Anaesthesiol Scand*	OR/ICU	cardiac or aortic aneurysm surgery	20	0.31 (0.87)	-1.40	2.00	na
Lefrant	1995	*Can J Anaesth*	ICU	critically ill	19	0.80 (1.20)	-1.60	3.20	na
Böttiger	1996	*Intensive Care Med*	ICU	cardiac surgery	22	0.05 (0.56)	-1.05	1.15	na
Jacquet	1996	*Intensive Care Med*	ICU	cardiac surgery or critically ill (septic shock)	23	-0.01 (0.69)	-1.39	1.37	22.3
Le Tulzo	1996	*J Clin Monit*	ICU	critically ill	21	0.39 (0.85)	-1.28	2.06	21.7
Böttiger (A)	1997	*Anaesthesia*	OR	liver transplantation (different time points)	12	0.24 (1.79)	-3.34	3.82	na
Böttiger (B)	1997	*Anaesthesia*	OR	liver transplantation (different time points)	12	-0.79 (4.31)	-9.41	7.84	na
Boyle	1997	*Aust Crit Care*	ICU	critically ill	38	0.07 (0.85)	-1.63	1.77	na
Burchell	1997	*Crit Care Med*	ICU	critically ill	21	-0.49 (1.01)	-2.51	1.53	na
Greim (A)	1997	*Anesth Analg*	OR	liver transplantation (infusion rate ≤ 1000 mL/h)	14	0.20 (0.90)	-1.60	2.00	na
Greim (B)	1997	*Anesth Analg*	OR	liver transplantation (infusion rate > 1000 mL/h)	14	1.00 (1.80)	-2.60	4.60	na
Lazor	1997	*J Cardiothorac Vasc Anesth*	OR/ICU	cardiac surgery or liver transplantation	29	-0.06 (0.49)	-1.04	0.92	na
Mihm	1998	*Crit Care Med*	ICU	critically ill	47	-0.12 (0.84)	-1.80	1.56	na
Monchi	1998	*J Crit Care*	ICU	critically ill (cardiogenic or septic shock)	19	-0.12 (0.92)	-1.90	1.70	na
Rödig	1998	*Eur J Anaesthesiol*	OR	cardiac surgery	24	0.13 (0.52)	-0.90	1.16	19.1
Seguin	1998	*Can J Anaesth*	ICU	critically ill	15	0.00 (0.74)	-1.45	1.45	22.7
Albert	1999	*Am J Crit Care*	ICU	critically ill	60	-0.74 (2.20)	-5.14	3.66	89.3
Cathelyn	1999	*J Cardiovasc Nurs*	ICU	cardiac surgery	7	0.39 (0.37)	-0.35	1.13	na
Neto (A)	1999	*Crit Care Med*	ICU	cardiac surgery (different PACs)	22	-0.07 (0.53)	-1.12	0.99	na

Neto (B)	1999	*Crit Care Med*	ICU	cardiac surgery (different PACs)	22	-0.02 (0.49)	-1.00	0.97	na
Rödig (A)	1999	*Br J Anaesth*	OR/ICU	cardiac surgery (ejection fraction > 45%)	13	0.12 (0.72)	-1.33	1.54	24.1
Rödig (B)	1999	*Br J Anaesth*	OR/ICU	cardiac surgery (ejection fraction < 45%)	13	0.06 (0.76)	-1.45	1.57	25.5
Schmid (A)	1999	*Intensive Care Med*	ICU	cardiac or aortic aneurysm surgery (different software versions and fluid bolus temperatures)	14	0.00 (0.90)	-1.80	1.80	na
Schmid (B)	1999	*Intensive Care Med*	ICU	cardiac or aortic aneurysm surgery (different software versions and fluid bolus temperatures)	14	0.20 (0.80)	-1.40	1.80	na
Schmid (C)	1999	*Intensive Care Med*	ICU	cardiac or aortic aneurysm surgery (different software versions and fluid bolus temperatures)	14	0.36 (0.84)	-1.32	2.04	na
Schmid (D)	1999	*Intensive Care Med*	ICU	cardiac or aortic aneurysm surgery (different software versions and fluid bolus temperatures)	14	0.07 (0.70)	-1.33	1.47	na
Zöllner	1999	*Crit Care Med*	ICU	cardiac surgery	20	-0.52 (1.29)	-3.10	2.06	na
Sakka	2000	*J Cardiothorac Vasc Anesth*	ICU	critically ill (sepsis or septic shock)	12	0.43 (0.71)	-0.96	1.82	na
Nelson	2001	*Crit Care Med*	ICU	critically ill (surgical)	28	-0.11 (0.74)	-1.56	1.34	18.7
Zöllner (A)	2001	*Can J Anaesth*	ICU	cardiac surgery (different PACs)	9	-0.09 (1.04)	-2.17	1.99	27.2
Zöllner (B)	2001	*Can J Anaesth*	ICU	cardiac surgery (different PACs)	10	-0.04 (0.74)	-1.52	1.44	25.8
Della Rocca	2002	*Br J Anaesth*	OR	liver transplantation	62	0.02 (0.74)	-1.46	1.50	19.0
Rauch	2002	*Acta Anaesthesiol Scand*	OR/ICU	cardiac surgery	25	-0.40 (1.25)	-2.85	2.05	na
Singh	2002	*J Cardiothorac Vasc Anesth*	OR	cardiac surgery	20	-0.10 (0.73)	-1.52	1.33	na
Della Rocca	2003	*Can J Anaesth*	OR	lung transplantation	58	0.15 (0.70)	-1.24	1.54	22.3
Kotake	2003	*Anesthesiology*	OR	aortic aneurysm surgery	28	0.38 (1.17)	-1.91	2.67	47.2
Mielck	2003	*J Cardiothorac Vasc Anesth*	ICU	cardiac surgery	22	0.28 (0.94)	-1.60	2.16	29.0
Padua	2003	*Minerva Anestesiol*	OR/ICU	cardiac surgery	16	-0.02 (0.11)	-0.24	0.20	4.8
Thierry	2003	*Intensive Care Med*	ICU	cardiac surgery	8	0.07 (0.66)	-1.25	1.39	na
Ishihara	2004	*J Clin Monit Comput*	ICU	cardiac surgery	36	-0.64 (0.84)	-2.32	1.04	na
Leather	2004	*Anaesthesia*	OR	cardiac surgery	34	0.40 (1.05)	-1.70	2.50	56.9
Bendjelid	2006	*Intensive Care Med*	ICU	cardiac surgery	14	0.33 (0.60)	-0.87	1.58	22.0
Button	2007	*Br J Anaesth*	OR/ICU	cardiac surgery	31	0.33 (1.19)	-2.05	2.72	na
Manecke	2007	*J Cardiothorac Vasc Anesth*	ICU	cardiac surgery	50	0.50 (0.66)	-0.82	1.82	na
McGee	2007	*Crit Care*	ICU	critically ill	84	0.66 (1.05)	-1.43	2.76	na

Bao	2008	*Hepatobiliary Pancreat Dis Int*	OR	liver transplantation	13	-0.18 (0.97)	-2.09	1.73	22.0
Costa (A)	2008	*Intensive Care Med*	ICU	liver transplantation (CO < 8 L/min)	23	-0.52 (1.19)	-2.89	1.85	18.7
Costa (B)	2008	*Intensive Care Med*	ICU	liver transplantation (CO > 8 L/min)	23	0.44 (1.36)	-2.27	3.15	14.5
Kotake	2009	*J Clin Monit Comput*	OR	aortic aneurysm surgery	42	0.19 (0.81)	-1.43	1.81	33.4
Cecconi	2010	*Minerva Anestesiol*	ICU	critically ill	29	0.30 (1.00)	-1.70	2.20	28.0
Hamm	2010	*Anaesth Intensive Care*	OR/ICU	cardiac surgery	9	0.03 (0.42)	-0.79	0.85	17.0
Akiyoshi	2011	*J Anesth*	OR	liver transplantation	20	0.79 (1.55)	-2.25	3.83	43.0
Cecchini	2012	*Annu Int Conf IEEE Eng Med Biol Soc*	ICU	cardiac surgery	15	0.27 (0.48)	-0.68	1.21	23.9
Costa	2014	*J Cardiothorac Vasc Anesth*	ICU	liver transplantation	20	-0.68 (1.31)	-3.29	1.93	29.9
Peyton	2014	*Anaesth Intensive Care*	OR	cardiac surgery or liver transplantation	30	0.10 (1.50)	-2.84	3.04	51.3
Cho	2016	*J Clin Monit Comput*	OR	cardiac surgery	20	-0.38 (0.57)	-1.50	0.74	26.7
Ganter	2016	*J Clin Monit Comput*	ICU	critically ill (septic shock)	47	0.23 (1.30)	-2.32	2.78	40.4

A, B, C, or D refer to different subsections of a study that has been treated as two or as four studies in the analysis. CO, cardiac output; ICU, intensive care unit; MD, mean of the differences; n, number of patients; na, not available; OR, operating room; PAC, pulmonary artery catheter; PE, percentage error; SD, standard deviation; 95%-LOA, 95%-limits of agreement.
