# Supplementary material for: Agreement between continuous and intermittent pulmonary artery thermodilution for cardiac output measurement in perioperative and intensive care medicine: a systematic review and meta-analysis
Source: Crit Care. 2021 Mar 29;25:125. doi: 10.1186/s13054-021-03523-7 (PMC8006374; doi:10.1186/s13054-021-03523-7)
Supplement: Supplementary file 4 — Additional file 4. Risk of bias assessment. This file contains the results of the risk of bias assessment of all included studies. [file 13054_2021_3523_MOESM4_ESM.pdf]

Additional file 4: Risk of bias assessment

	Risk of Bias				Applicability concerns		
	Patient Selection	Index Test	Reference Standard	Flow and Timing	Patient Selection	Index Test	Reference Standard
Yelderman 1992	?	✗	✗	✓	✓	✓	✓
Boldt 1994	✓	✓	✓	✓	✓	✓	✓
Hogue 1994	✓	✓	✓	✓	✓	✓	✓
Böttiger 1995	✓	✓	✓	✓	✓	✓	✓
Ditmyer 1995	✓	✓	✓	✓	✓	✓	✓
Haller 1995	✓	✓	✓	✓	✓	✓	✓
Jakobsen 1995	?	✓	✓	✓	✓	✓	✓
Lefrant 1995	✓	✓	✓	✓	✓	✓	✓
Böttiger 1996	✓	✓	✓	✓	✓	✓	✓
Jacquet 1996	✓	✓	✓	?	✓	✓	✓
Le Tulzo 1996	✓	✓	✓	?	✓	✓	✓
Böttiger 1997	✓	✓	✓	✓	✓	✓	✓
Boyle 1997	✗	✗	✓	✗	✓	✓	✓
Burchell 1997	✗	✓	✓	✗	✓	✓	✓
Greim 1997	✓	✓	✓	✓	✓	✓	✓
Lazor 1997	✓	✓	✓	✓	✓	✓	✓
Mihm 1998	✓	✓	✓	✓	✓	✓	✓
Monchi 1998	✓	✓	✓	✓	✓	✓	✓
Rödig 1998	✓	✓	✓	✓	✓	✓	✓
Seguin 1998	✓	✓	✓	✓	✓	✓	✓
Albert 1999	✓	✓	✓	✓	✓	✓	✓
Cathelyn 1999	✓	✓	✓	?	✓	✓	✓
Neto 1999	✓	✓	✓	✓	✓	✓	✓
Rödig 1999	✓	✓	✓	✓	✓	✓	✓
Schmid 1999	✓	✓	✓	✓	✓	✓	✓
Zöllner 1999	✓	✓	✓	✓	✓	✓	✓
Sakka 2000	✓	✓	✓	✓	✓	✓	✓
Nelson 2001	✓	✓	✓	✓	✓	✓	✓
Zöllner 2001	✓	✓	✓	?	✓	✓	✓
Della Rocca 2002	✓	✓	✓	✓	✓	✓	✓
Rauch 2002	✓	✓	✓	✓	✓	✓	✓
Singh 2002	✓	✓	✓	✓	✓	✓	✓
Della Rocca 2003	✗	?	✓	?	✓	✓	✓
Kotake 2003	✓	?	✓	?	✓	✓	✓
Mielck 2003	✓	✓	✓	✓	✓	✓	✓
Padua 2003	✓	✓	✓	?	✓	✓	✓
Thierry 2003	✗	✓	✗	✓	✓	✓	✓
Ishihara 2004	✓	✓	✓	✓	✓	✓	✓
Leather 2004	✓	✓	✓	✓	✓	✓	✓
Bendjelid 2006	✓	✓	✓	✓	✓	✓	✓
Button 2007	✓	✓	✓	✓	✓	✓	✓
Manecke 2007	✓	✓	✓	✓	✓	✓	✓
McGee 2007	✓	?	✓	✓	✓	✓	✓
Bao 2008	✓	✓	✓	?	✓	✓	✓
Costa 2008	✓	✓	✓	?	✓	✓	✓
Kotake 2009	✓	✓	✓	✓	✓	✓	✓
Cecconi 2010	✓	✓	?	✓	✓	✓	✓
Hamm 2010	✓	?	?	?	✓	✓	✓
Akiyoshi 2011	✓	✓	✓	✓	✓	✓	✓
Cecchini 2012	✗	✗	✗	✗	✓	✓	✓
Costa 2014	✓	✓	✓	✓	✓	✓	✓
Peyton 2014	✓	?	?	✓	✓	✓	✓
Cho 2016	✓	✓	✓	✓	✓	✓	✓
Ganter 2016	✓	✓	✓	✓	✓	✓	✓

QUADAS-2: Quality Assessment of Diagnostic Accuracy Studies

✓ = low risk ✗ = high risk ? = unclear risk
