# Supplementary figures and images for: Agreement between continuous and intermittent pulmonary artery thermodilution for cardiac output measurement in perioperative and intensive care medicine: a systematic review and meta-analysis
Source: Crit Care. 2021 Mar 29;25:125. doi: 10.1186/s13054-021-03523-7 (PMC8006374; doi:10.1186/s13054-021-03523-7)

**Additional file 5: Funnel plot for cardiac output with Eggers regression test**

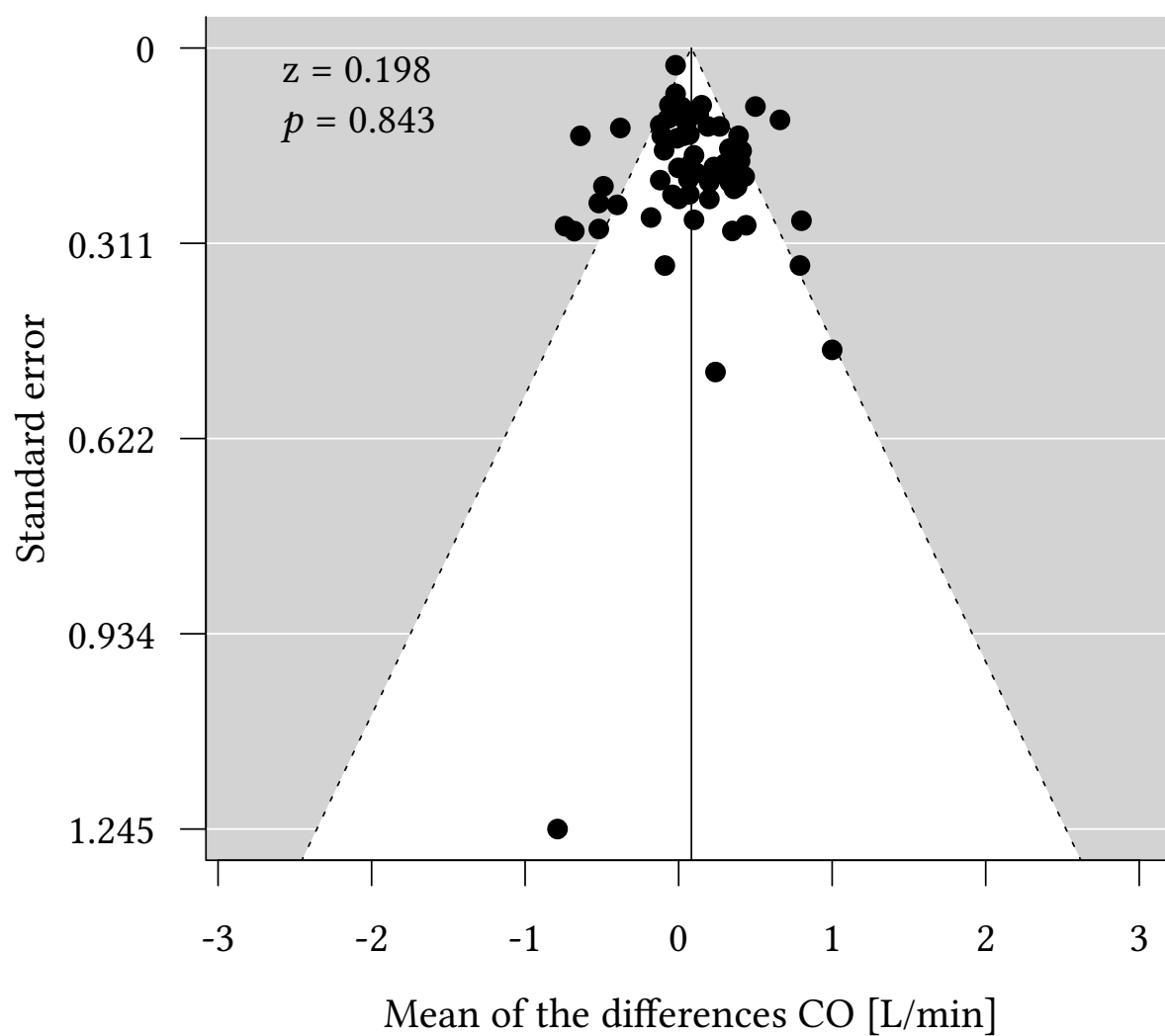

Supplement: Supplementary file 5 — Additional file 5. Funnel plot for cardiac output with Eggers regression test. Funnel plot indicating the risk of publication bias across studies including Eggers regression for cardiac output (CO). [file 13054_2021_3523_MOESM5_ESM.pdf]

**Additional file 6: Funnel plot for the percentage error with Eggers regression test**

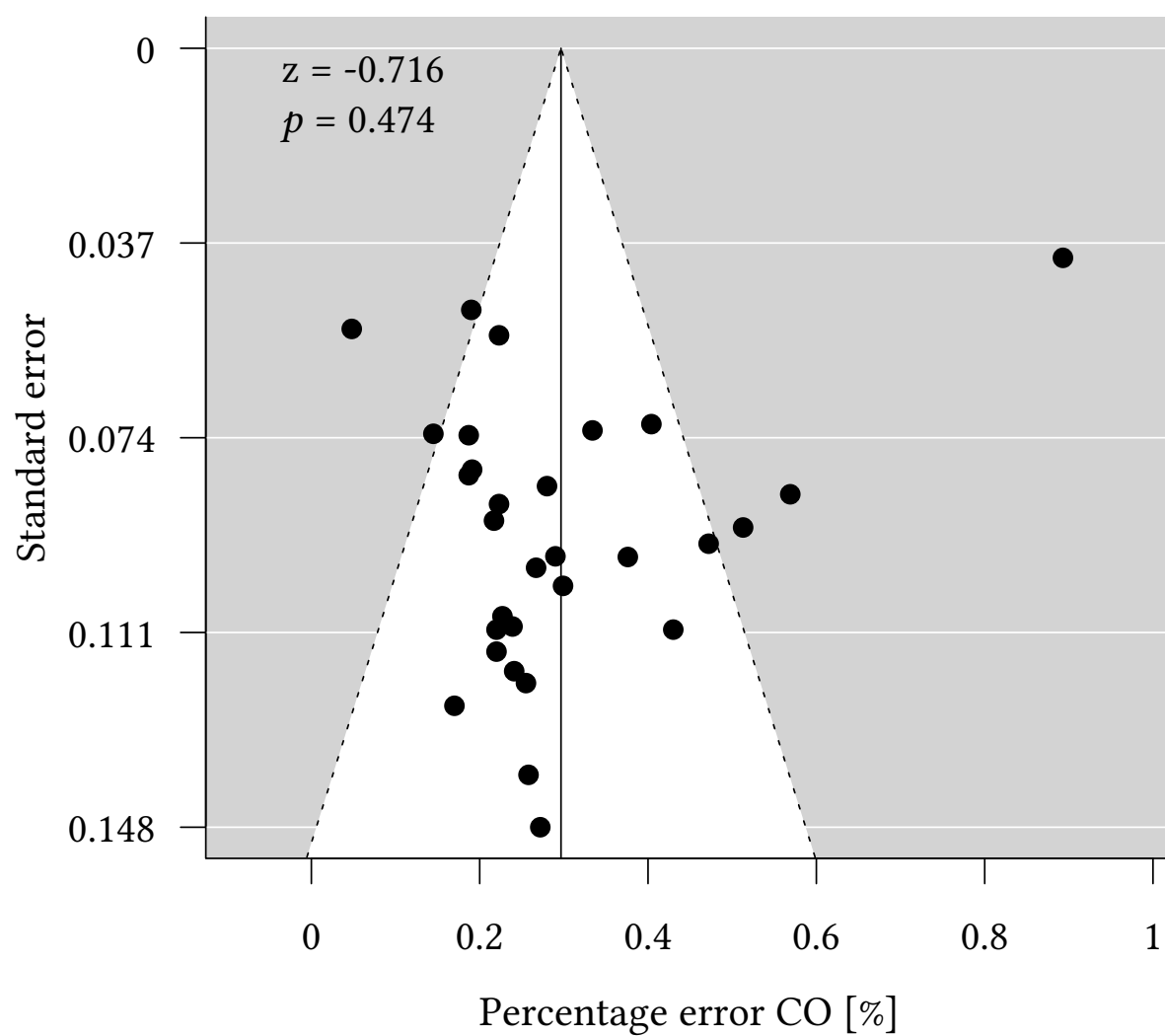

Supplement: Supplementary file 6 — Additional file 6. Funnel plot for the percentage error with Eggers regression test. Funnel plot indicating the risk of publication bias across studies including Eggers regression for the percentage error. CO, cardiac output. [file 13054_2021_3523_MOESM6_ESM.pdf]

### Additional file 13: Influence of the level of cardiac output

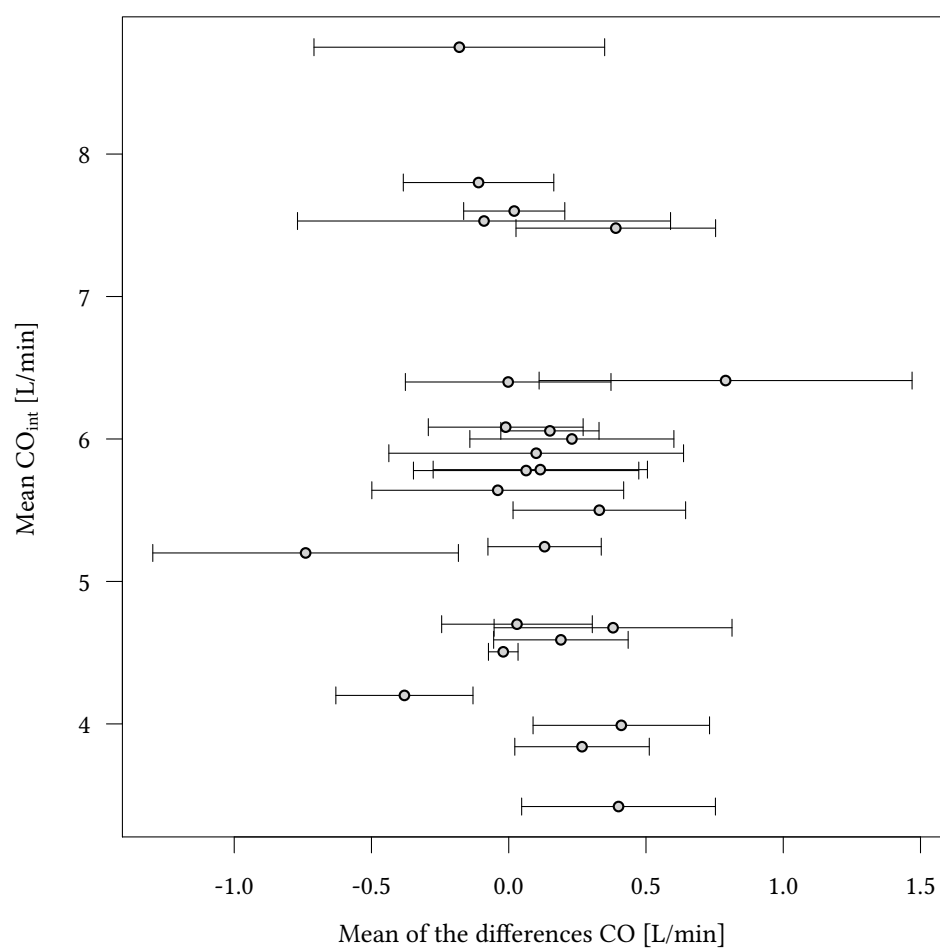

Supplement: Supplementary file 13 — Additional file 13. Influence of the level of cardiac output. Plot showing the relation between the mean of the differences (dots) with corresponding 95%-confidence interval (bars) per individual study and mean intermittent pulmonary artery catheter-derived cardiac output (CO) measurement (COint). [file 13054_2021_3523_MOESM13_ESM.pdf]

## Additional file 14: Influence of the year of publication

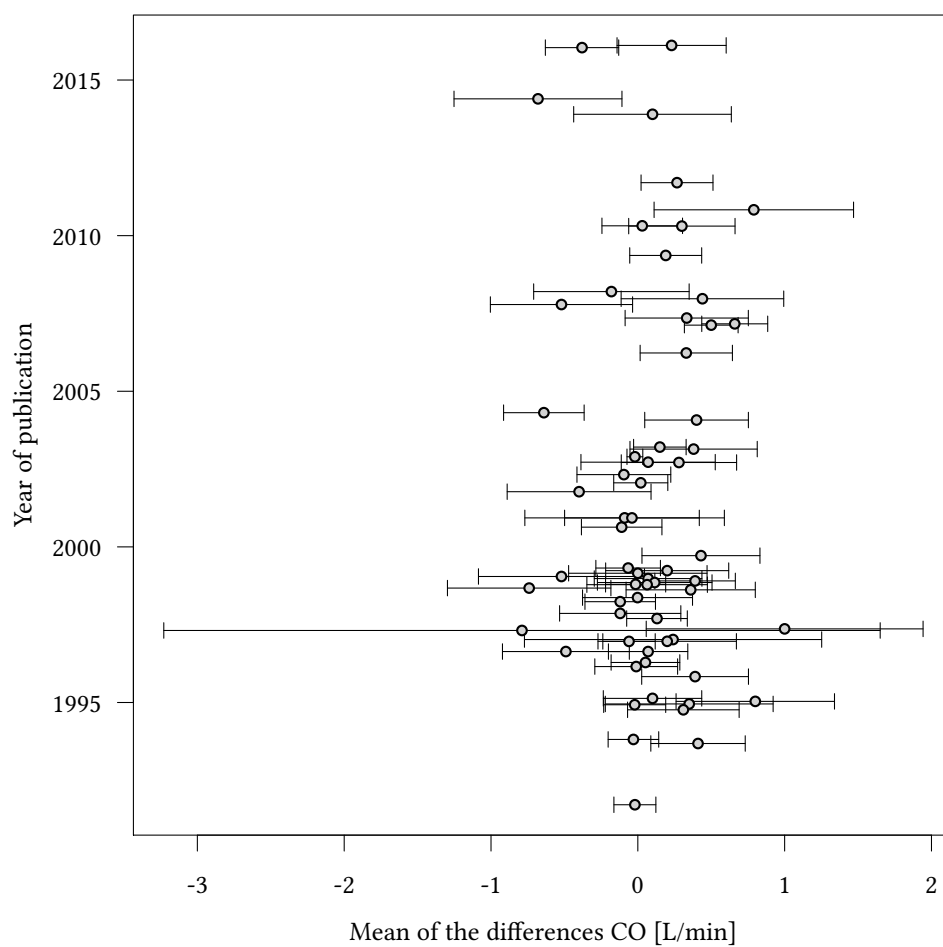

Supplement: Supplementary file 14 — Additional file 14. Influence of the year of publication. Plot showing the relation between the mean of the differences (dots) with corresponding 95%-confidence interval (bars) per individual study and the year of publication. CO, cardiac output. [file 13054_2021_3523_MOESM14_ESM.pdf]
