# Supplementary material for: Agreement between continuous and intermittent pulmonary artery thermodilution for cardiac output measurement in perioperative and intensive care medicine: a systematic review and meta-analysis
Source: Crit Care. 2021 Mar 29;25:125. doi: 10.1186/s13054-021-03523-7 (PMC8006374; doi:10.1186/s13054-021-03523-7)
Supplement: Supplementary file 8 — Additional file 8. Forest plot showing subgroup analysis for the setting “intensive care unit”. Forest plot showing the results of the subgroup analysis for the setting “intensive care unit” for cardiac output (CO) with mean of the differences (dots) and corresponding 95%-confidence interval (bars) per individual study in relation to the overall random effects model-derived pooled estimate (vertical dashed line). Heterogeneity is presented with Cochran’s Q and I2. N, number of patients per study. Costa and colleagues [36], Neto and colleagues [60], and Zöllner and colleagues [72] are treated as two studies in the analysis (A and B). Schmid and colleagues [67] is treated as four studies in the analysis (A, B, C, and D). [file 13054_2021_3523_MOESM8_ESM.pdf]

Additional file 8: Forest plot showing subgroup analysis for the setting “intensive care unit”

Study	N	Mean of the differences	(95%-confidence interval)
Albert (1999)	60	-0.74	(-1.30, -0.18)
Costa (2014)	20	-0.68	(-1.25, -0.11)
Ishihara (2004)	36	-0.64	(-0.91, -0.37)
Zöllner (1999)	20	-0.52	(-1.09, 0.05)
Costa (2008) A	23	-0.52	(-1.00, -0.04)
Burchell (1997)	21	-0.49	(-0.92, -0.06)
Mihm (1998)	47	-0.12	(-0.36, 0.12)
Monchi (1998)	19	-0.12	(-0.53, 0.29)
Nelson (2001)	28	-0.11	(-0.38, 0.16)
Zöllner (2001) A	9	-0.09	(-0.77, 0.59)
Neto (1999) A	22	-0.07	(-0.29, 0.15)
Zöllner (2001) B	10	-0.04	(-0.50, 0.42)
Boldt (1994)	35	-0.03	(-0.20, 0.14)
Yelderman (1992)	54	-0.02	(-0.16, 0.12)
Neto (1999) B	22	-0.01	(-0.22, 0.19)
Jacquet (1996)	23	-0.01	(-0.29, 0.27)
Seguin (1998)	15	-0.00	(-0.38, 0.37)
Schmid (1999) A	14	0.00	(-0.47, 0.47)
Böttiger (1996)	22	0.05	(-0.18, 0.29)
Thierry (2003)	8	0.07	(-0.39, 0.53)
Schmid (1999) D	14	0.07	(-0.30, 0.44)
Boyle (1997)	38	0.07	(-0.20, 0.34)
Ditmyer (1995)	14	0.10	(-0.24, 0.44)
Schmid (1999) B	14	0.20	(-0.22, 0.62)
Ganter (2016)	47	0.23	(-0.14, 0.60)
Cecchini (2012)	15	0.27	(0.02, 0.51)
Mielck (2003)	22	0.28	(-0.11, 0.67)
Cecconi (2010)	29	0.30	(-0.06, 0.66)
Bendjelid (2006)	14	0.33	(0.02, 0.64)
Haller (1995)	12	0.35	(-0.22, 0.92)
Schmid (1999) C	14	0.36	(-0.08, 0.80)
Cathelyn (1999)	7	0.39	(0.12, 0.66)
Le Tulzo (1996)	21	0.39	(0.03, 0.75)
Sakka (2000)	12	0.43	(0.03, 0.83)
Costa (2008) B	23	0.44	(-0.11, 0.99)
Manecke (2007)	50	0.50	(0.32, 0.68)
McGee (2007)	84	0.66	(0.44, 0.88)
Lefrant (1995)	19	0.80	(0.26, 1.34)
Random effects model for all<br>Heterogeneity: Q = 146.5 (*P* <0.001)<br>I^2 = 76%		0.07	(-0.04, 0.17)

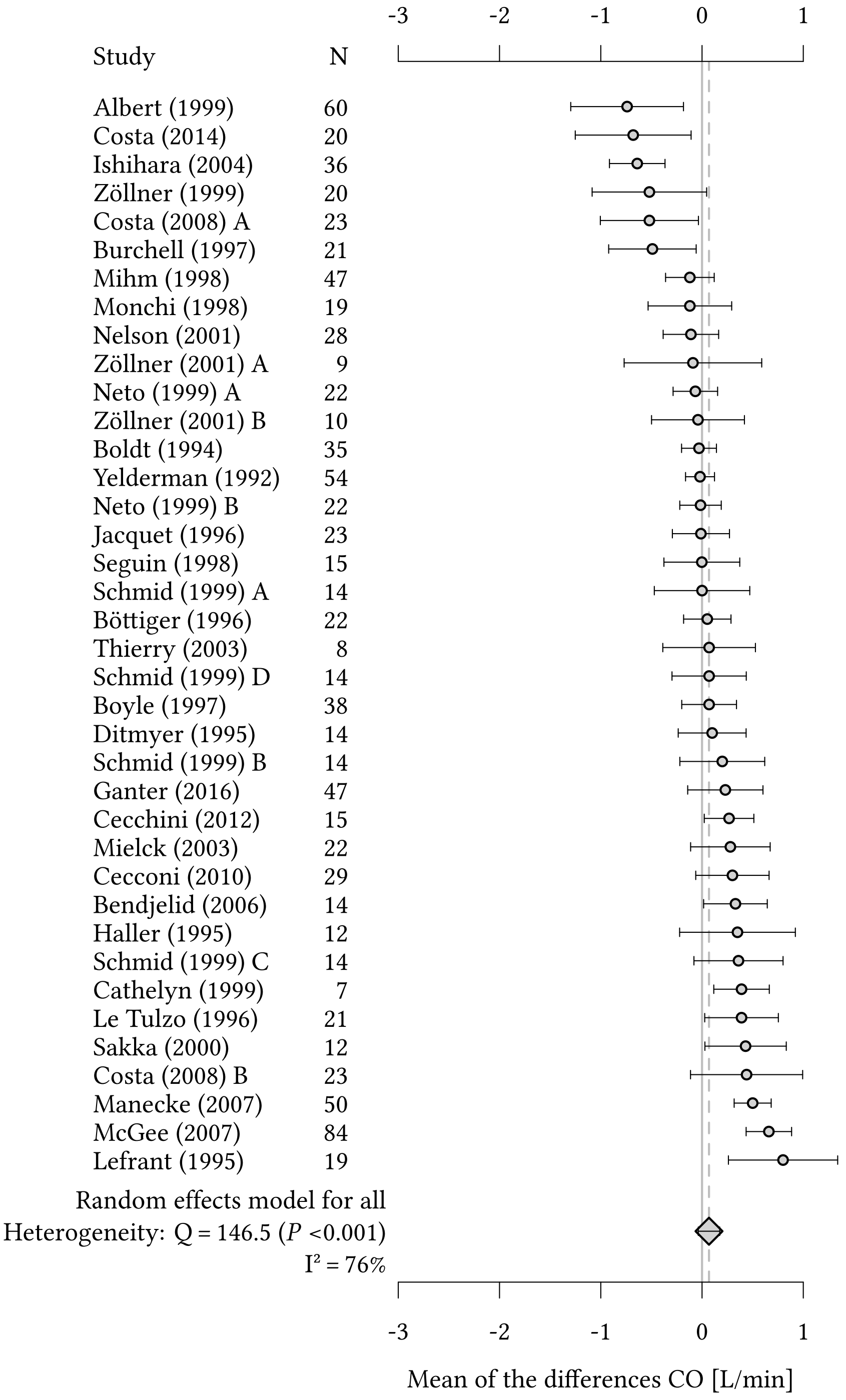

Pooled 95%-limits of agreement: -1.66 to 1.76 L/min
