# Supplementary material for: Agreement between continuous and intermittent pulmonary artery thermodilution for cardiac output measurement in perioperative and intensive care medicine: a systematic review and meta-analysis
Source: Crit Care. 2021 Mar 29;25:125. doi: 10.1186/s13054-021-03523-7 (PMC8006374; doi:10.1186/s13054-021-03523-7)
Supplement: Supplementary file 11 — Additional file 11. Forest plot showing subgroup analysis for studies with reported or calculable percentage error. Forest plot showing the results of the subgroup analysis for studies with reported or calculable percentage error for cardiac output (CO) with mean of the differences (dots) and corresponding 95%-confidence interval (bars) per individual study in relation to the overall random effects model-derived pooled estimate (vertical dashed line). Heterogeneity is presented with Cochran’s Q and I2. N, number of patients per study. Costa and colleagues [36], Rödig and colleagues [65], and Zöllner and colleagues [72] are treated as two studies in the analysis (A and B). [file 13054_2021_3523_MOESM11_ESM.pdf]

Additional file 11: Forest plot showing subgroup analysis for studies with reported or calculable percentage error

Study	N	Mean of the differences	(95%-confidence interval)
Albert (1999)	60	-0.74	(-1.30, -0.18)
Costa (2014)	20	-0.68	(-1.25, -0.11)
Costa (2008) A	23	-0.52	(-1.00, -0.04)
Cho (2016)	20	-0.38	(-0.63, -0.13)
Bao (2008)	13	-0.18	(-0.71, 0.35)
Nelson (2001)	28	-0.11	(-0.38, 0.16)
Zöllner (2001) A	9	-0.09	(-0.77, 0.59)
Zöllner (2001) B	10	-0.04	(-0.50, 0.42)
Padua (2003)	16	-0.02	(-0.07, 0.03)
Jacquet (1996)	23	-0.01	(-0.29, 0.27)
Seguin (1998)	15	-0.00	(-0.38, 0.37)
Della Rocca (2002)	62	0.02	(-0.16, 0.20)
Hamm (2010)	9	0.03	(-0.24, 0.30)
Rödig (1999) B	13	0.06	(-0.35, 0.47)
Peyton (2014)	30	0.10	(-0.44, 0.64)
Rödig (1999) A	13	0.12	(-0.28, 0.51)
Rödig (1998)	24	0.13	(-0.08, 0.34)
Della Rocca (2003)	58	0.15	(-0.03, 0.33)
Kotake (2009)	42	0.19	(-0.05, 0.43)
Ganter (2016)	47	0.23	(-0.14, 0.60)
Cecchini (2012)	15	0.27	(0.02, 0.51)
Mielck (2003)	22	0.28	(-0.11, 0.67)
Cecconi (2010)	29	0.30	(-0.06, 0.66)
Bendjelid (2006)	14	0.33	(0.02, 0.64)
Kotake (2003)	28	0.38	(-0.05, 0.81)
Le Tulzo (1996)	21	0.39	(0.03, 0.75)
Leather (2004)	34	0.40	(0.05, 0.75)
Hogue (1994)	25	0.41	(0.09, 0.73)
Costa (2008) B	23	0.44	(-0.11, 0.99)
Akiyoshi (2011)	20	0.79	(0.11, 1.47)
Random effects model for all<br>Heterogeneity: Q = 73.0 (P <0.001)<br>I^2 = 66%		0.08	(-0.01, 0.18)

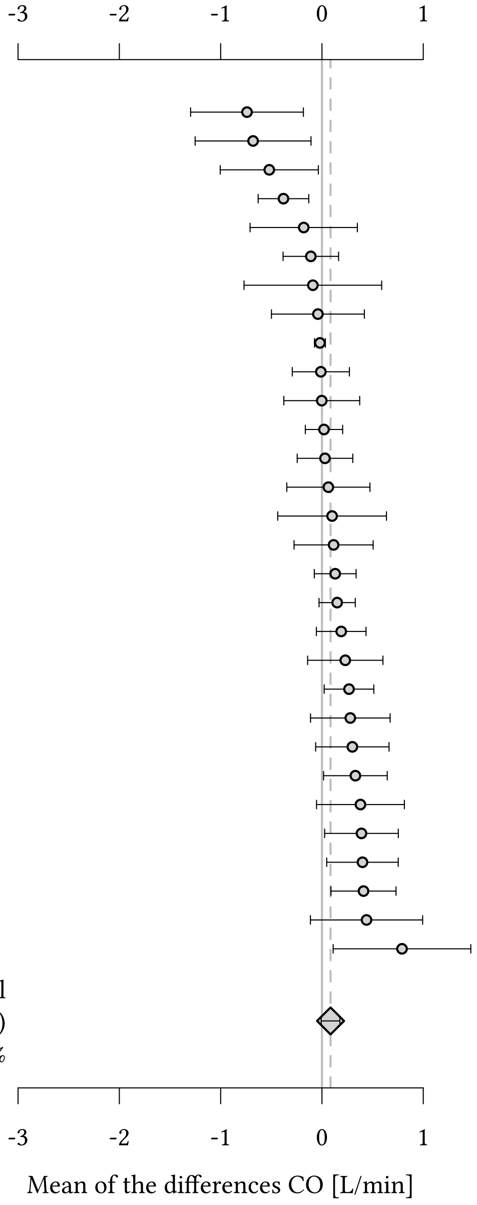

Pooled 95%-limits of agreement: -1.73 to 1.87 L/min
