# Supplementary material for: Agreement between continuous and intermittent pulmonary artery thermodilution for cardiac output measurement in perioperative and intensive care medicine: a systematic review and meta-analysis
Source: Crit Care. 2021 Mar 29;25:125. doi: 10.1186/s13054-021-03523-7 (PMC8006374; doi:10.1186/s13054-021-03523-7)
Supplement: Supplementary file 12 — Additional file 12. Forest plot showing subgroup analysis for studies without reported or calculable percentage error. Forest plot showing the results of the subgroup analysis for studies without reported or calculable percentage error for cardiac output (CO) with mean of the differences (dots) and corresponding 95%-confidence interval (bars) per individual study in relation to the overall random effects model-derived pooled estimate (vertical dashed line). Heterogeneity is presented with Cochran’s Q and I2. N, number of patients per study. Böttiger and colleagues [26], Greim and colleagues [41], and Neto and colleagues [60], are treated as two studies in the analysis (A and B). Schmid and colleagues [67] is treated as four studies in the analysis (A, B, C, and D). [file 13054_2021_3523_MOESM12_ESM.pdf]

## Additional file 12: Forest plot showing subgroup analysis for studies without reported or calculable percentage error

| Study | N | Mean of the differences | (95%-confidence interval) |
|---|---|---|---|
| Böttiger (1997) B | 12 | -0.79 | (-3.23, 1.65) |
| Ishihara (2004) | 36 | -0.64 | (-0.91, -0.37) |
| Zöllner (1999) | 20 | -0.52 | (-1.09, 0.05) |
| Burchell (1997) | 21 | -0.49 | (-0.92, -0.06) |
| Rauch (2002) | 25 | -0.40 | (-0.89, 0.09) |
| Mihm (1998) | 47 | -0.12 | (-0.36, 0.12) |
| Monchi (1998) | 19 | -0.12 | (-0.53, 0.29) |
| Singh (2002) | 20 | -0.10 | (-0.41, 0.22) |
| Neto (1999) A | 22 | -0.07 | (-0.29, 0.15) |
| Lazor (1997) | 29 | -0.06 | (-0.24, 0.12) |
| Boldt (1994) | 35 | -0.03 | (-0.20, 0.14) |
| Böttiger (1995) | 30 | -0.02 | (-0.23, 0.19) |
| Yelderman (1992) | 54 | -0.02 | (-0.16, 0.12) |
| Neto (1999) B | 22 | -0.01 | (-0.22, 0.19) |
| Schmid (1999) A | 14 | 0.00 | (-0.47, 0.47) |
| Böttiger (1996) | 22 | 0.05 | (-0.18, 0.29) |
| Thierry (2003) | 8 | 0.07 | (-0.39, 0.53) |
| Schmid (1999) D | 14 | 0.07 | (-0.30, 0.44) |
| Boyle (1997) | 38 | 0.07 | (-0.20, 0.34) |
| Ditmyer (1995) | 14 | 0.10 | (-0.24, 0.44) |
| Schmid (1999) B | 14 | 0.20 | (-0.22, 0.62) |
| Greim (1997) A | 14 | 0.20 | (-0.27, 0.67) |
| Böttiger (1997) A | 12 | 0.24 | (-0.77, 1.25) |
| Jakobsen (1995) | 20 | 0.31 | (-0.07, 0.69) |
| Button (2007) | 31 | 0.33 | (-0.09, 0.75) |
| Haller (1995) | 12 | 0.35 | (-0.22, 0.92) |
| Schmid (1999) C | 14 | 0.36 | (-0.08, 0.80) |
| Cathelyn (1999) | 7 | 0.39 | (0.12, 0.66) |
| Sakka (2000) | 12 | 0.43 | (0.03, 0.83) |
| Manecke (2007) | 50 | 0.50 | (0.32, 0.68) |
| McGee (2007) | 84 | 0.66 | (0.44, 0.88) |
| Lefrant (1995) | 19 | 0.80 | (0.26, 1.34) |
| Greim (1997) B | 14 | 1.00 | (0.06, 1.94) |
| Random effects model for all<br>Heterogeneity: Q = 125.7 (*P* <0.001)<br>$I^2$ = 76% | | 0.08 | (-0.03, 0.20) |

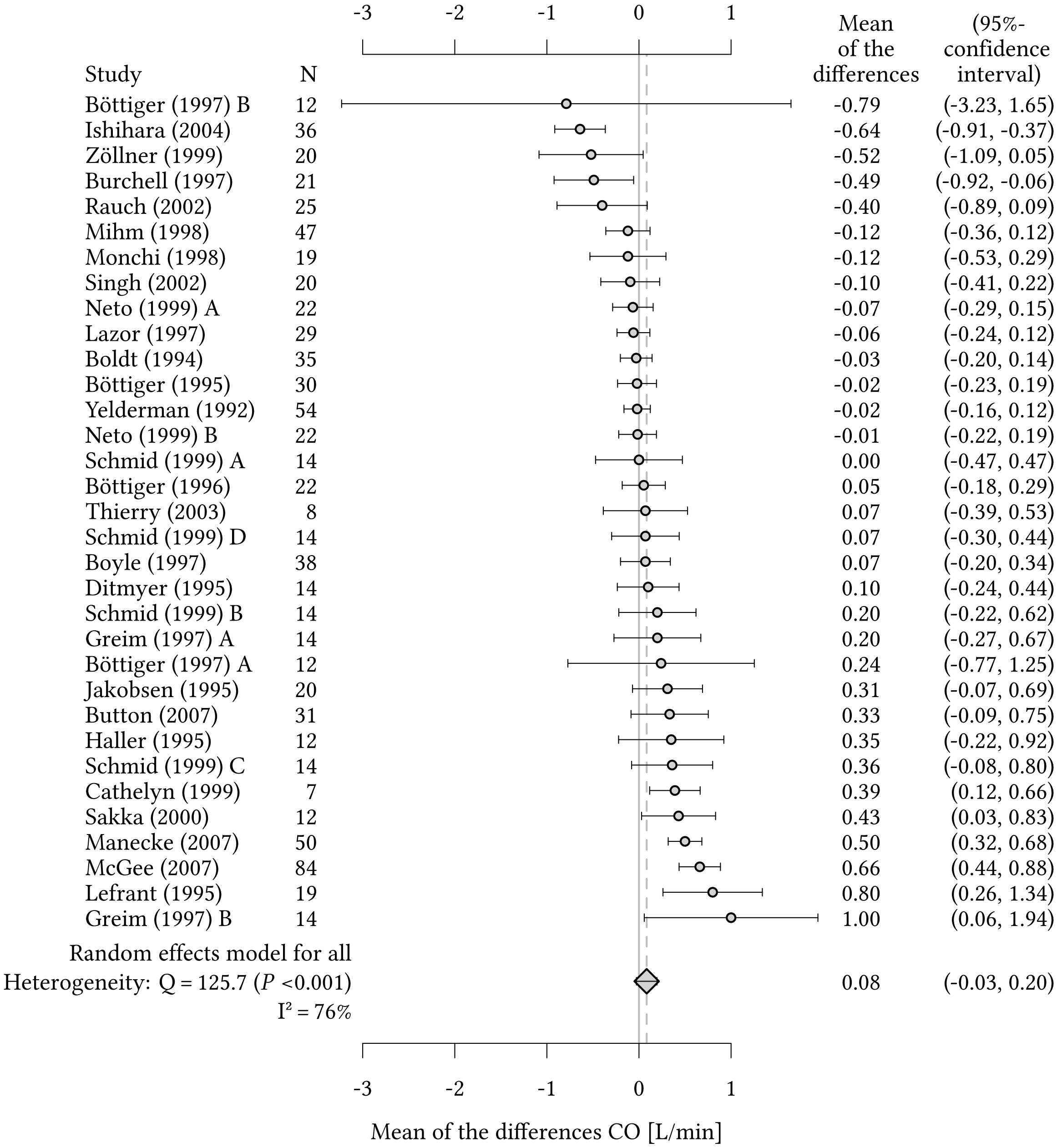


Pooled 95%-limits of agreement: -1.63 to 1.83 L/min
